# Supplementary material for: Causal Relationship between Aging and Anorexia Nervosa: A White-Matter-Microstructure-Mediated Mendelian Randomization Analysis
Source: Biomedicines. 2024 Aug 16;12(8):1874. doi: 10.3390/biomedicines12081874 (PMC11351342; doi:10.3390/biomedicines12081874)
Supplement: Supplementary file 1 [file biomedicines-12-01874-s001.zip › Supplementary Figure/Figure S8/Leaveoneout plot of MR analysis for TL to ubm-b-1678.pdf]

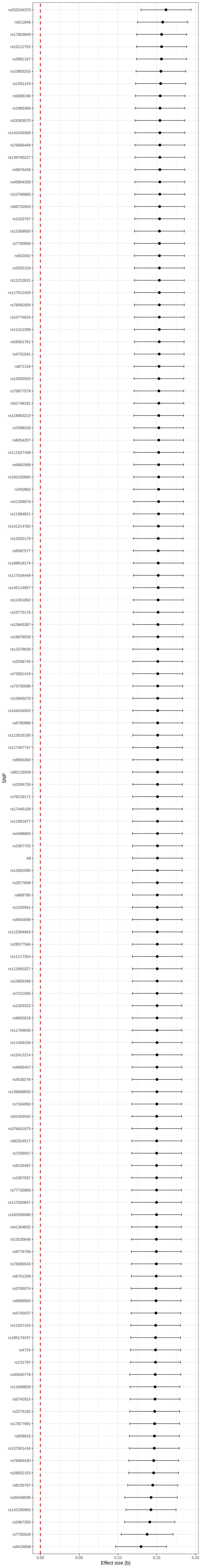

rs202034370
rs611646
rs17803849
rs10112752
rs3891167
rs10905255
rs1291143
rs9398196
rs1985369
rs28363070
rs142426306
rs76666449
rs139795227
rs9878436
rs45604339
rs10768683
rs66731853
rs1023767
rs12369950
rs7790856
rs932002
rs2555104
rs11212631
rs117512405
rs78491606
rs10774624
rs11412296
rs56061761
rs4731541
rs871134
rs12925933
rs79977579
rs61748181
rs116863223
rs7099229
rs6054257
rs111527438
rs6881568
rs150150565
rs450962
rs41269079
rs11584821
rs141214782
rs12932179
rs6587577
rs188918174
rs117034449
rs145114957
rs12451892
rs10773176
rs10845387
rs16978028
rs11579626
rs2538745
rs73581419
rs73730598
rs10840270
rs144204502
rs6790988
rs113525195
rs117407747
rs9955360
rs80116508
rs2056726
rs76219171
rs17445108
rs11991877
rs4498805
rs1907702
All
rs13062095
rs2977608
rs869785
rs1332941
rs9940099
rs112394943
rs28577594
rs11117354
rs111950327
rs10805346
rs7221585
rs1003322
rs9600019
rs11769630
rs11426156
rs12412214
rs4695407
rs4530278
rs139669835
rs7164950
rs61405042
rs376641875
rs80324517
rs7209057
rs8102497
rs1957937
rs77732866
rs117630647
rs182059586
rs41304832
rs13230646
rs6776756
rs76065543
rs6751209
rs3785074
rs6669563
rs4743037
rs11557154
rs185174247
rs4724
rs131797
rs35640778
rs11699829
rs5742915
rs2276182
rs17677991
rs939916
rs137901416
rs75664430
rs28502153
rs8105767
rs35446936
rs143190905
rs2967355
rs7705526
rs9419958
SNP
0.00
0.05
0.10
0.15
0.20
Effect size (b)
